# High expression of PTPN21 in B-cell non-Hodgkin's gastric lymphoma, a positive mediator of STAT5 activity

**DOI:** 10.1038/bcj.2015.107

**Published:** 2016-01-15

**Authors:** J H C Plani-Lam, T C Chow, Y-H Fan, B Garcia-Bloj, L Cheng, D Y Jin, W Hancock, S Fanayan, E Ingley, Y-Q Song

**Affiliations:** 1 School of Biomedical Sciences (formally known as Department of Biochemistry), Li Ka Shing Faculty of Medicine, University of Hong Kong, Hong Kong, China; 2 Cancer and Cell Biology Division, Harry Perkins Institute of Medical Research and Centre for Medical Research, The University of Western Australia, Nedlands, Western Australia, Australia; 3 Laboratory of Brain and Cognitive Sciences (State Key Laboratory), University of Hong Kong, Hong Kong, China; 4 Barnett Institute, Department of Chemistry and Chemical Biology, Northeastern University, Boston, MA, USA; 5 Department of Biomedical Sciences, Macquarie University, Sydney, New South Wales, Australia; 6 Centre for Genomic Sciences, University of Hong Kong, Hong Kong, China

Investigations of specific pathologic mechanisms of a disease often provide extraordinary opportunities to identify suitable diagnostic markers and alternative treatment options. We investigated whether altered protein tyrosine phosphatase (PTP) activity might be linked to the development of stomach cancer, which is the most common gastrointestinal malignancy and the third leading cause of cancer-related death worldwide.^1^

Perturbations of PTP activity caused by mutations and manifested as PTP overexpression result in malignant transformation. Thus, PTPs might serve as alternative diagnostic markers and anticancer targets. Here, we report an uncommon phosphatase, PTP non-receptor 21 (PTPN21), which in recent years has been suspected of playing a pathologic role in gastrointestinal tract tumorigenesis. A previous mutational analysis of the *PTP* gene superfamily identified PTPN21 as having the highest mutation frequency in types of colon cancer with microsatellite instability,^2^ and a later study found that PTPN21 affects mitogenic signaling in bladder cancer.^3^ Coincidentally, a systematic expression profiling study of 87 PTPs^4^ and an independent oligonucleotide microarray study,^5^ identified 22 PTPs (including PTPN21)^4^ and PTPN21,^5^ respectively, both studies illustrated distinctly different expression patterns of PTPN21 in gastric carcinoma and normal gastric tissue.

Our analysis of PTPN21 expression in samples of stomach tissue obtained from 56 individuals revealed low to medium expression of PTPN21 in samples of human gastric non-carcinoma tissue (*n*=10; Figure 1a), as well as samples of TNM Classification of Malignant Tumors stage I (*n*=4), II (*n*=10) and III (*n*=23) human gastric adenocarcinoma tissue. A subgroup analysis of human stage I gastric carcinoma tissue revealed that PTPN21 expression was significantly associated with cytosolic E-cadherin expression (*r*=0.40; *P*-value<0.001; Supplementary Figure S1 and Supplementary Methods and Materials). On a proportional scale, this suggested an association between PTPN21 and E-cadherin intensities (*r*=0.24; *P*-value<0.07). A *χ^2^*-test revealed a significant correlation (*P*-value=0.01) between PTPN21 levels and the expression scores for cytosolic E-cadherin, suggesting an inverse relationship between the expression patterns of membrane E-cadherin and PTPN21. However, overall PTPN21 is often expressed at moderate, but largely uniform, levels in virtually all noncancerous gastric tissues.

Physiologically, PTPN21 directly dephosphorylates ErbB1 and ErbB4 (members of the receptor tyrosine kinase family),^3, 6^ resulting in upregulation of its downstream signaling. In turn, this results in the constitutive activation of Src and increases the activity of ETS domain-containing protein (Elk-1) via regulation of the MEK-Elk-1 signaling pathway.^7^ However, not much is known about PTPN21 and its alternative ErbB4-dependent downstream targets. Hence, the present study extends our previous work, which demonstrated that PTPN21 controls the accumulation of ErbB4 in human embryonic kidney cells and mouse embryonic cortical neurons.^6^ In the present study, reciprocal immunoprecipitation confirmed that PTPN21 interacts with ErbB4 in SGC 7901 gastric cancer cells (Supplementary Figure S2 and Supplementary Methods and Materials) in a manner previously described.^6^

Moreover, ErbB4 is a receptor that is directly upstream of STAT5,^8^ and STAT5 activation mediates a wide variety of activities induced by growth factors, cytokines and interferons. For example, STAT5 is activated by epidermal growth factor (EGF), which induces translocation of STAT5 into the nucleus and is highly expressed in both lymphoma cell lines and clinical samples of human lymphoma gastrointestinal tissue.^9^ In addition, STAT5 binds to the gamma-interferon activation motif within the promoter region of STAT5 target genes to activate transcription of the pro-survival genes, *β-casein* and *Cyclin D1*.^10^ Such findings suggest that PTPN21 expression can induce higher than normal levels of STAT5 activity in both human B-cell lymphoma and gastric cells.

To examine the functional consequences of the suggested association between PTPN21 and STAT5, we analyzed the effect of PTPN21 on the STAT5 target gene *β-casein*. PTPN21 promoted dose-dependent increases (up to 3.1±0.32-fold) in the transcription of *β-casein*, and this induction was further enhanced 5.2±0.43-fold by co-expression of ErbB4 with PTPN21 (Figure 1b and Supplementary Methods and Materials) in a STAT5A-specific manner (Figure 1c). Immunoreactivity assays showed that in SGC 7901 cells, STAT5 levels increased with binding activity in a PTPN21-dependent manner (Figures 1d and e). The ErbB receptor antagonist AG1478 blocked all PTPN21-dependent *β-casein* transcription, suggesting that the effect of PTPN21 on *β-casein* is dependent on the ErbB4 receptor.

Unexpectedly, PTPN21 failed to enhance β-casein translation, even following EGF stimulation. This discrepancy in translation of Cyclin D1 and β-casein may be due to differences in the availability of specific coactivators required to assemble the transcriptional coactivator complexes (TCC) for either protein. For example, it is important to note that STAT5 interacts with Oct1 to form a TCC needed to promote *Cyclin D1* transcription.^10^ In addition, STAT5 must also bind to nuclear receptor coactivator 1 (NCOA1) and the glucocorticoid receptor (GR) to form a TCC that promotes *β-Casein* transcription.^11^ This suggests that the *β-casein* transcription rates are also dependent on the formation of a transcription factor complex with NCOA1 and GR; the transcription reporter assay used in this study accurately reflected the significance of PTPN21-dependent STAT5 activation.

Ligand stimulation in HEK 293 cells overexpressing PTPN21 resulted in elevated phosphorylation of STAT5 (Supplementary Figure S3A; HEK 293), when compared with cells that were either not treated with an agonist or transfected with the phosphatase-dead PTPN21 (PD_PTPN21). Expression of Cyclin D1 (another STAT5 target) was also evaluated, and PTPN21 expression was positively correlated with Cyclin D1 translation in both HEK 293 and SGC 7901 cells (Supplementary Figure S3B). PTPN21-dependent STAT5 activity was either absent or only slightly detectable in non-B-cell lymphoma EL4 cells (Supplementary Figure S3C). Adding to this complexity, information obtained from an open-access PrESSTo data set collected by the Fantom consortium^12^ described elevated levels of PTPN21 promoter binding in hematologic cells, but not B cells (Supplementary Figure S3D). It is important to note that although this study might have identified a mechanistic role for PTPN21 in gastric cell lines, the lymphoma cell line stimulated with EGF showed only a slight induction of STAT5 activity, suggesting that the erythropoietin receptor remained the key transmembrane receptor for activation of STAT5 in the malignant hematopoietic cell line. Taken together, these data suggest the existence of cross talk between gastric stroma and adjacent malignant lymphoma cells, triggered by an extrinsic signaling transduction cross talk mechanism that remains to be elucidated. As a result, we further investigated the role of PTPN21 in gastric lymphoma by conducting histopathological analyses of human gastric B cells obtained from the tissue of patients with either gastric B-cell Hodgkin's lymphoma (HL; n=6) or non-Hodgkin's lymphoma (NHL; n=6).

Gastric B cells from NHL patients contained significantly increased levels of PTPN21; however, when based on intensity and proportional scales, only moderately increased levels of PTPN21 were found in B cells from HL patients, and low levels of PTPN21 in noncancerous tissues (*P*-value<0.05; Mann–Whitney *U*; Figure 2). Lower expression of membrane E-cadherin was correlated with a higher expression of PTPN21 in human gastric B cells obtained from NHL and HL patients. In contrast, expression of cytosolic E-cadherin was positively correlated with PTPN21 expression in human gastric adenocarcinoma cells. Additional analyses of the influence of age and gender were performed using a fitting linear model and Mann–Whitney *U* model respectively, and the results showed no correlation between PTPN21 expression and age or gender. These results suggest that elevated PTPN21 expression is both abnormal and involved in the pathogenesis of gastric B-cell NHL and HL. Overall, based on the expression profiles of PTPN21 in human gastric tissues, it is clear that PTPN21 plays a less important pathologic role in adenocarcinoma than in B-cell NHL.

In addition, elevated PTPN21 levels were positively correlated with cytosolic levels of the carcinoma marker E-cadherin. Coincidentally, Pez, the Drosophila homolog of PTPN21 that colocalizes with membrane-bound E-cadherin in basolateral membranes, is known to be essential for the epithelial-mesenchymal transition process.^13^ Overexpression of Pez has been shown to inhibit cell–cell contact and abolish localization of E-cadherin to the plasma membrane.^13^ Such findings support those in the present study, which showed that significantly increased PTPN21 expression in malignant human gastric lymphoma tissue was associated with reduced E-cadherin levels on the cell membrane (Figure 2).

In summary, PTPN21 was found to be the most mutated PTP in colorectal tumors with microsatellite instability; however, its role in human malignancies remains unclear. The data presented in this report show that PTPN21 is highly expressed in human gastric B-cell NHL tissue, and promotes STAT5 activity. In addition, moderate levels of PTPN21 expression were identified in samples of non-carcinoma gastric tissue. Consequently, our data suggest a role for PTPN21 in regulating STAT5 activity, and implicate PTPN21 in the tumorigenic process leading to development of hematologic-related human gastric carcinoma.

## Figures and Tables

**Figure 1:**
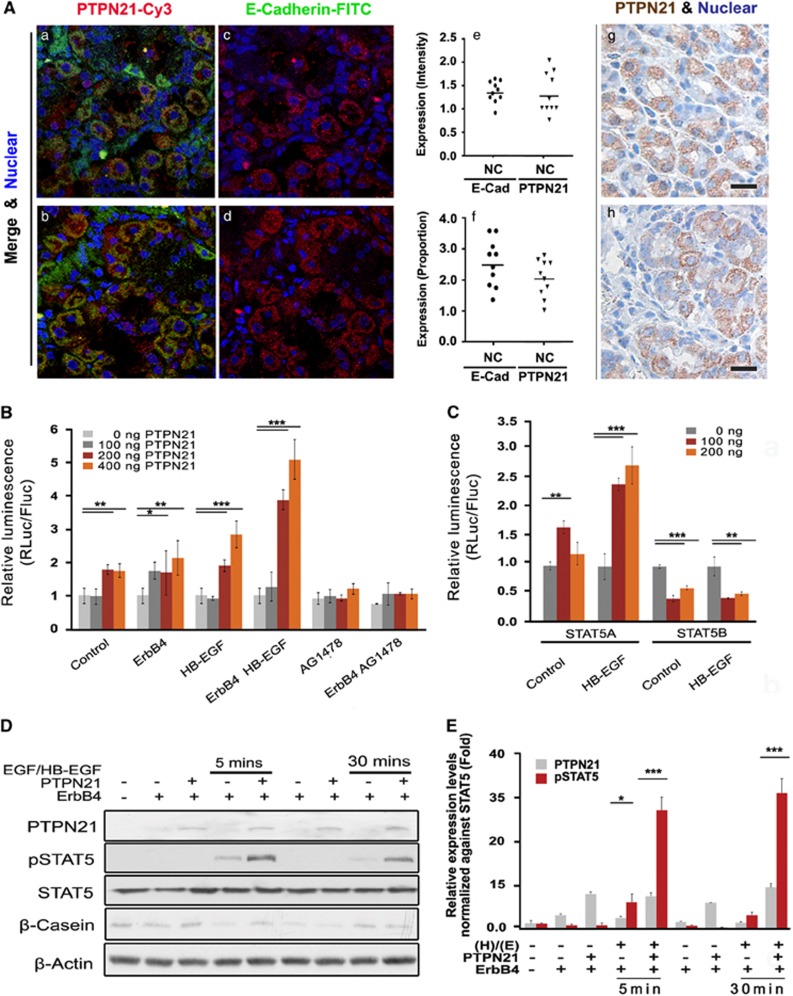
(**A**) (a–f) Noncancerous human gastric tissues were stained with anti-PTPN21, and then incubated with Cy3-conjugated secondary antibody or anti-E-Cadherin antibody. After staining, the samples were incubated with FITC-conjugated secondary antibody to visualize membrane-localized E-cadherin and total PTPN21 expression. (g–h) Immunohistochemical staining with 3,3′-diaminobenzidine (DAB; brown) identified total PTPN21 expression, and hematoxylin (blue) was used as a nuclear counter stain. Scale bar indicates 20 μm. (**B**) Binding activity of *β-casein* promoter-dependent STAT5 obtained from EGF/HB-EGF treated cells was promoted when translating a firefly luciferase (FLuc) reporter containing PTPN21, but not when translating a control renilla luciferase (RLuc) reporter. The ErbB4 antagonist AG1478 impaired STAT5 binding activity. The light gray bar indicates cells transfected with empty vector and used as a control. Other bars represent cells transfected with pcDNA_PTPN21 plasmid in a dose-dependent manner (100–400 ng). Cells were co-transfected with ErbB4 and/or treated with EGF/HB-EGF (50 ng/ml) or AG1478 (5–10 μm), as indicated. (**C**) Constitutive STAT5A promoted *β-casein* promoter-dependent STAT5 binding activity, whereas constitutive STAT5B failed to promote high levels of *β-casein* promoter-dependent STAT5 binding activity. (**D**, **E**) SGC 7901 cells were co-transfected with ErbB4 and PTPN21 or phosphatase-dead mutant PD_PTPN21 for 36–48 h, and then treated with EGF/HB-EGF (50 ng/ml) for 0, 5 or 30 min as indicated. Immunoblotting analysis revealed enhanced STAT5 activity after transfection with PTPN21, which manifested as increased phospho (T694)-STAT5 levels. β-Casein expression levels were also analyzed. Total STAT5 protein expression and β-actin housekeeping protein were used to ensure comparable total protein levels when analyzing different samples. For all tests, a significant *P*<0.05 is represented with *, a *P*<0.01 is represented with ** and *P*<0.001 is represented with ***. Error bars represent the standard deviation.

**Figure 2:**
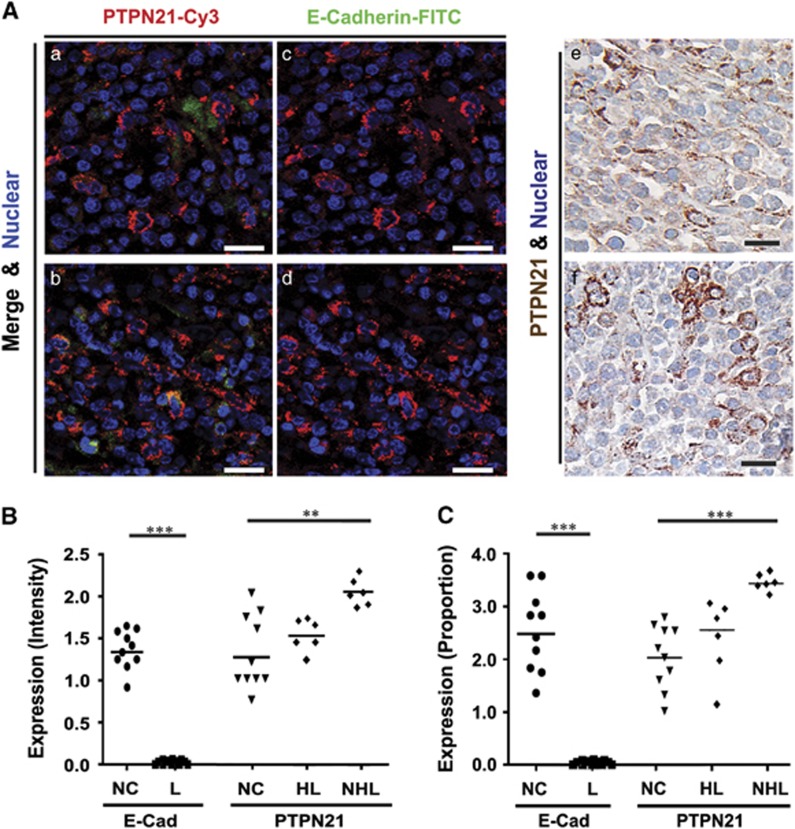
(**A**) PTPN21 is overexpressed in human gastric B-cells non-Hodgkin's lymphoma tissue. (a–f) Malignant human gastric B-cells non-Hodgkin's lymphoma. (a–d) Fluorescence immunohistochemistry using anti-PTPN21 and Cy3-conjugated secondary antibody or anti-E-cadherin antibody and FITC-conjugated secondary antibody revealed membrane-localized E-cadherin and total PTPN21 expression. (e–f) Immunohistochemical staining with 3,3′-diaminobenzidine (DAB; brown) identified total PTPN21 expression, with hematoxylin (blue) being used as a nuclear counter stain. (**B**) Intensity and (**C**) proportion scores for PTPN21 and E-cadherin expression in noncancerous human gastric tissues (NC; *n* =10), malignant non-Hodgkin's (NHL; *n*=6) and Hodgkin's lymphoma tissue (HL; *n*=6). NHL and HL samples represented by L. Scale bar indicates 20 μm. For all tests, a significant *P*<0.05 is represented with *, a *P*<0.01 is represented with ** and *P*<0.001 is represented with ***. Error bars represent the standard deviation.

## References

[bib1] 1Guzicka-Kazimierczak R, Zdziarska B, Kazimierczak A, Sledz M. Gastric non-Hodgkin's lymphoma—clinical symptoms and diagnostic problems. Wiad Lek 2011; 64: 3–8. 21812356

[bib2] 2Korff S, Woerner SM, Yuan YP, Bork P, von Knebel Doeberitz M, Gebert J. Frameshift mutations in coding repeats of protein tyrosine phosphatase genes in colorectal tumors with microsatellite instability. BMC Cancer 2008; 8: 329. 19000305 10.1186/1471-2407-8-329 PMC2586028

[bib3] 3Carlucci A, Porpora M, Garbi C, Galgani M, Santoriello M, Mascolo M et al. PTPD1 supports receptor stability and mitogenic signaling in bladder cancer cells. J Biol Chem 2010; 285: 39260–39270. 20923765 10.1074/jbc.M110.174706 PMC2998146

[bib4] 4Wu CW, Kao HL, Li AF, Chi CW, Lin WC. Protein tyrosine-phosphatase expression profiling in gastric cancer tissues. Cancer Lett 2006; 242: 95–103. 16338072 10.1016/j.canlet.2005.10.046

[bib5] 5Hippo Y, Taniguchi H, Tsutsumi S, Machida N, Chong JM, Fukayama M et al. Global gene expression analysis of gastric cancer by oligonucleotide microarrays. Cancer Res 2002; 62: 233–240. 11782383

[bib6] 6Plani-Lam JH, Chow TC, Siu KL, Chau WH, Ng MH, Bao S et al. PTPN21 exerts pro-neuronal survival and neuritic elongation via ErbB4/NRG3 signaling. Int J Biochem Cell Biol 2015; 11: 53–62. 10.1016/j.biocel.2015.02.003 25681686

[bib7] 7Cardone L, Carlucci A, Affaitati A, Livigni A, DeCristofaro T, Garbi C et al. Mitochondrial AKAP121 binds and targets protein tyrosine phosphatase D1, a novel positive regulator of src signaling. Mol Cell Biol 2004; 24: 4613–4626. 15143158 10.1128/MCB.24.11.4613-4626.2004 PMC416429

[bib8] 8Jones FE, Welte T, Fu XY, Stern DF. ErbB4 signaling in the mammary gland is required for lobuloalveolar development and Stat5 activation during lactation. J Cell Biol 1999; 147: 77–88. 10508857 10.1083/jcb.147.1.77 PMC2164978

[bib9] 9Ebi M, Kataoka H, Shimura T, Hirata Y, Mizushima T, Mizoshita T et al. The role of neuregulin4 and HER4 in gastrointestinal malignant lymphoma. Mol Med Rep 2011; 4: 1151–1155. 21805036 10.3892/mmr.2011.542

[bib10] 10Magné S, Caron S, Charon M, Rouyez MC. Dusanter-Fourt I. STAT5 and Oct-1 form a stable complex that modulates cyclin D1 expression. Mol Cell Biol 2003; 23: 8934–8945. 14645506 10.1128/MCB.23.24.8934-8945.2003 PMC309603

[bib11] 11Chiba T, Kimura S, Takahashi K, Morimoto Y, Sanbe A, Ueda H et al. Serotonin suppresses β-casein expression via inhibition of the signal transducer and activator of transcription 5 (STAT5) protein phosphorylation in human mammary epithelial cells MCF-12A. Biol Pharm Bull 2014; 37: 1336–1340. 25087955 10.1248/bpb.b14-00273

[bib12] 12Andersson R, Gebhard C, Miguel-Escalada I, Hoof I, Bornholdt J, Boyd M et al. An atlas of active enhancers across human cell types and tissues. Nature 2014; 507: 455–461. 24670763 10.1038/nature12787 PMC5215096

[bib13] 13Wadham C, Gamble JR, Vadas MA, Khew-Goodall Y. The protein tyrosine phosphatase Pez is a major phosphatase of adherens junctions and dephosphorylates beta-catenin. Mol Biol Cell 2003; 14: 2520–2529. 12808048 10.1091/mbc.E02-09-0577 PMC194899

