# Supplementary figures and images for: High expression of PTPN21 in B-cell non-Hodgkin's gastric lymphoma, a positive mediator of STAT5 activity
Source: Blood Cancer J. 2016 Jan 15;6(1):e388–. doi: 10.1038/bcj.2015.107 (PMC4742624; doi:10.1038/bcj.2015.107)

Supplementary Figure 1

**A**

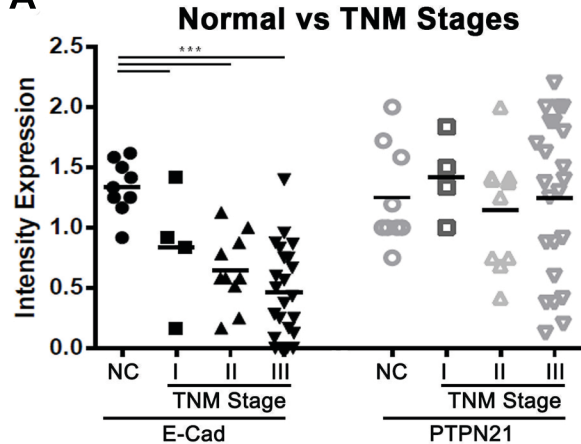

**B**

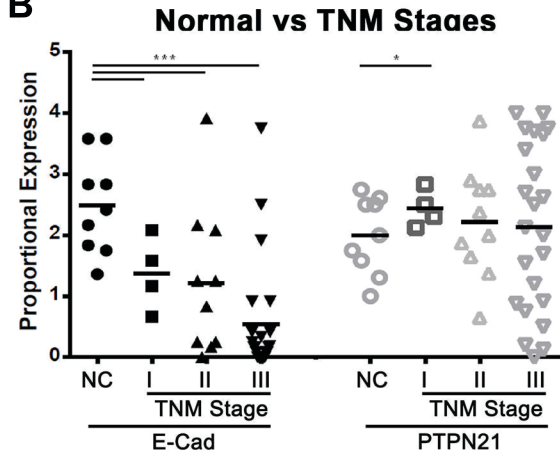

Supplement: Supplementary Figure 1 [file bcj2015107x1.pdf]

## Supplementary Figure 2

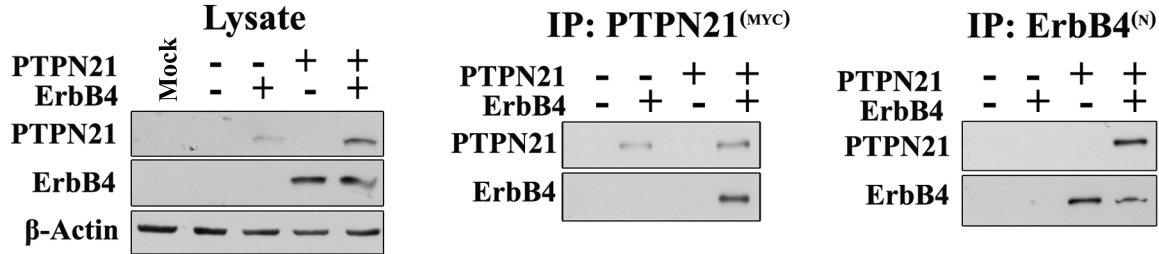

Supplement: Supplementary Figure 2 [file bcj2015107x2.pdf]

# Supplementary Figure 3

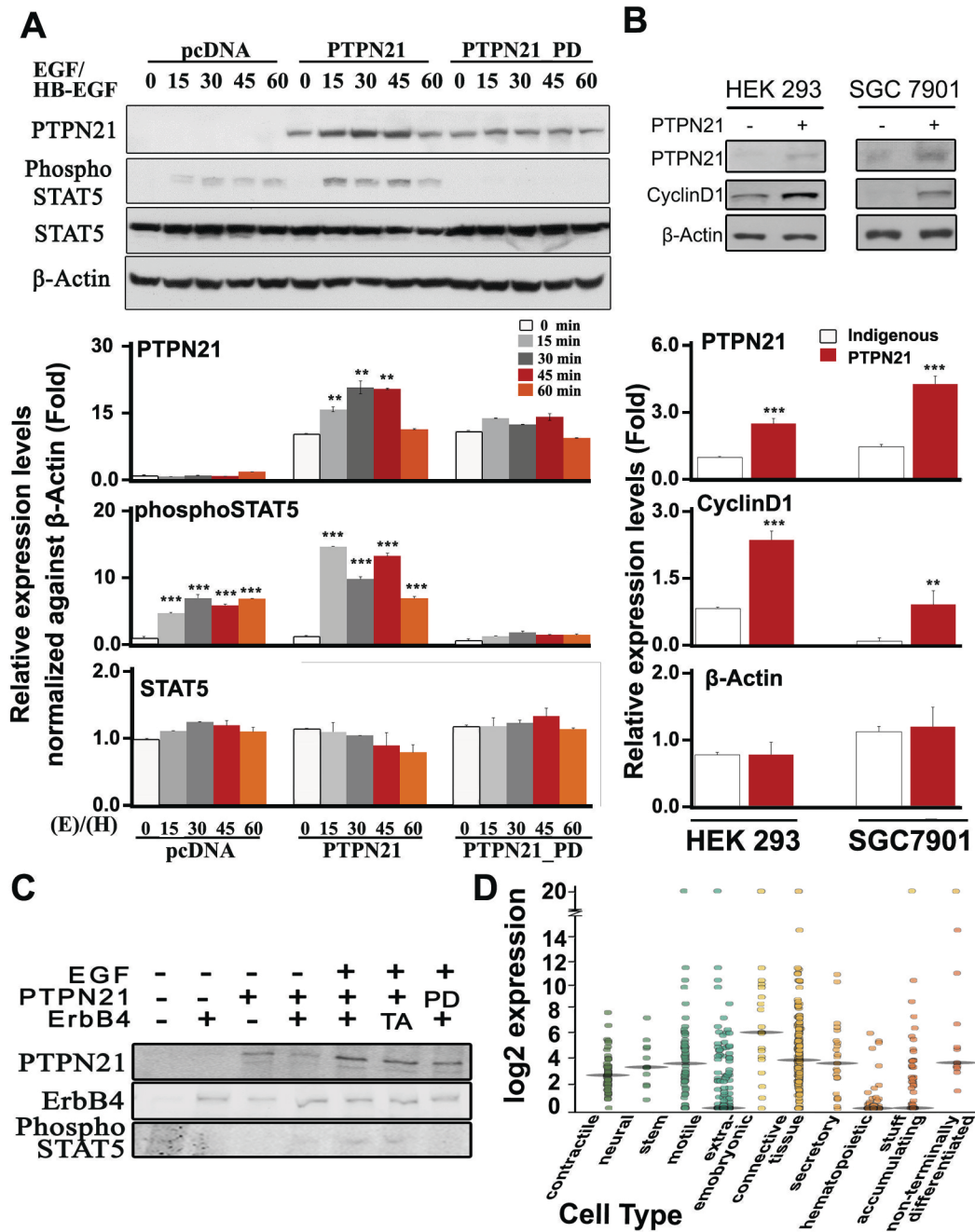

Supplement: Supplementary Figure 3 [file bcj2015107x3.pdf]
