# Supplementary material for: High expression of PTPN21 in B-cell non-Hodgkin's gastric lymphoma, a positive mediator of STAT5 activity
Source: Blood Cancer J. 2016 Jan 15;6(1):e388–. doi: 10.1038/bcj.2015.107 (PMC4742624; doi:10.1038/bcj.2015.107)
Supplement: Supplementary Figure Legends [file bcj2015107x4.docx]

**Supplementary Figure Legends**

**Supplementary Figure 1.** PTPN21 expression patterns in primary noncancerous gastric tissues and gastric adenocarcinoma tissues, representing 43 individual specimens. Staining intensities and proportional scores for PTPN21 and membrane E-cadherin. PTPN21 was moderately expressed in approximately 50% of noncancerous gastric tissues; however, PTPN21 showed expression varying from low to high in samples of gastric adenocarcinoma tissue representing various TNM stages.

**Supplementary Figure 2.** PTPN21 interacts with ErbB4. Reciprocal immunoprecipitation with an anti-myc epitope tag and anti-ErbB4 antibodies confirmed an interaction between PTPN21 and ErbB4. The reciprocal immunoprecipitation demonstrate that PTPN21 interacts with the ErbB4 receptor. The mock-transfection control; Transfected with vector only or transfected with PTPN21_myc and ErbB4 vectors, respectively. Input indicates expression of PTPN21 and ErbB4 in total cell lysates.

**Supplementary Figure 3.** SGC 7901 and HEK 293 cells were co-transfected with ErbB4 and PTPN21, or phosphatase-dead mutant PD_PTPN21 (PD) for 36-48 hours, and then treated with EGF/HB-EGF (50 ng/mL) for 0, 15, 30, 45 or 60 min, as indicated. Transfection with empty vector or pcDNA-PTPN21 produced the indicated PTPN21 and cyclinD1 protein levels in **A)** HEK 293, **B)** SGC 7901 and HEK 293. **C)** Transfection with empty vector, pcDNA-PTPN21, ErbB4, phosphatase-dead PTPN21 (PD) and/or ErbB4_1162A (TA), produced the indicated PTPN21 and phospho-STAT5 protein levels in lymphoma EL4 cell lines. **D)** Profiles of hematopoietic cell-type-specific PTPN21 transcripts. Box plots show the distribution of expression levels for the promoter as indicated by individual dots. Data were obtained from an open-access PrESSTo dataset collected by the Fantom consortium.[^39^](#_ENREF_39)
