# Supplementary material for: High expression of PTPN21 in B-cell non-Hodgkin's gastric lymphoma, a positive mediator of STAT5 activity
Source: Blood Cancer J. 2016 Jan 15;6(1):e388–. doi: 10.1038/bcj.2015.107 (PMC4742624; doi:10.1038/bcj.2015.107)
Supplement: Supplementary Materials and Methods [file bcj2015107x5.docx]

**Materials and Methods**

**Cell Culture**

SGC 7901 human gastric carcinoma cells, HEK 293 human embryonic kidney cells, Elk4 mouse lymphoma cells. All cell lines were actually obtained from the ATCC ® Essentials of Life Science Research Inc.

**Site-directed mutagenesis and transient transfection**

The plasmid pcDNA_PTPN21 was kindly provided by Dr. A. Feliciello (Università di Napoli).[^4^](#_ENREF_4)^,^[^28^](#_ENREF_28) Transfections were performed using Lipofectamine 2000 (Invitrogen; Carlsbad, CA, USA) or GeneJuice (Novagen; Madison, WI, USA) according to the manufacturer's protocol. Unless otherwise indicated, HEK293 human embryonic kidney cells (HEK 293) and human gastric cancer cells (SGC 7901) were first transfected and then serum starved overnight. A point mutation in the phosphatase domain (C1108S) and completely lack phosphatase activity, phosphatase-dead PTPN21 (PD_PTPN21) plasmid was constructed by site-directed mutagenesis (Millipore; Billerica, MA, USA) performed according to the manufacturer’s protocol and previous publications. [^4^](#_ENREF_4)^,^[^7^](#_ENREF_7)

**Immunoblotting and immunoprecipitation assays**

Lysates for use in immunoblotting or immunoprecipitation (IP) were obtained by using established protocols.[^7^](#_ENREF_7) Briefly, 1 mL of lysis buffer (50 mM Tris-HCl pH 7.5, 150 mM NaCl, 1% NP40, 0.5% sodium deoxycholate, 1% Triton X-100, 50 mM NaF, 100 mM PMSF, and 1 x Roche complete protease inhibitor cocktail) was added to each 60 mm culture dish. Next, cells were added to each dish and incubated in the lysis buffer for 20-30 min at 4°C. The cells were then centrifuged at 16,000 RCF, and the resulting supernatants were stored at -80°C. Immunoblotting was performed with the following primary antibodies; PTPN21 (ab12250, Abcam; Burlingame, CA, USA), β-actin (A5316, Sigma; St. Louis, MO, USA), β-casein (ab112595, Abcam), cyclin D1 (sc753, Santa Cruz Biotechnology; Santa Cruz, CA, USA), phospho694-STAT5 (D47E7, Cell Signaling Technology; Danvers, MA, USA), and STAT5 (sc835, Santa Cruz Biotechnology), followed by incubation with secondary antibodies (DAKO; Carpinteria, CA, USA). Staining was detected using enhanced chemiluminescence (ECL) substrate (GE Healthcare; Marlborough, MA, USA) followed by exposure to x-ray film (Kodak; Rochester, NY, USA).

**STAT5 binding activity in the luciferase assay**

The STAT5-luciferase (Luc) system was a kind gift from Dr. P. Koppikar (University of Pittsburgh, USA) and has been previously described.[^29^](#_ENREF_29) PTPN21 plasmids were transfected in a dose-dependent manner for 36 hours, and then treated with an ErbB4 inhibitor (AG1478; 5-10 μM) for one hour, followed by treatment with an ErbB agonist (EGF or HB-EGF; 50 ng/mL) for 5-30 min. The STAT5-dependent luciferase assay was performed according to the manufacturer's protocol (Promega) and is described in previous reports.[^29-31^](#_ENREF_29)

**Profiling of PTPN21 expression in human gastric lymphoma tissue and noncancerous gastric tissue by immunohistochemical staining**

Paraffin-embedded sections of 56 human gastric tissues (5-10 μm thick) were stained using immunohistochemical methods described in instructions provided by the manufacturer of the staining kit (Pantomics Inc; Richmond, CA, USA). Nine samples of noncancerous gastric tissue, 39 samples of gastric cancer tissue obtained from progressive grades of adenocarcinoma, and 12 samples of gastric lymphoma tissue were utilized to analyze the staining intensity and expression patterns of PTPN21 and the gastric cancer marker E-cadherin. Briefly, all sections were first treated with antigen retrieval buffer (DAKO), and then incubated with mouse anti-E-cadherin (ab1416, Abcam) and/or rabbit anti-PTPN21 antibodies. Slides with the stained tissue were mounted and examined using laser scanning confocal microscopy (Zeiss LSM710, Carl Zeiss Inc; Thornwood, NY, USA) or a Vector® DAB Substrate Kit (Vector Laboratories; Burlingame, CA, USA); images were taken at magnifications of ×200 to ×400. Due to regional variations in tissue specimens, three continuous microscopic views (×400 each) of each stained specimen were captured and recorded. Positive immunostaining was scored by two individual researchers using two different scoring systems as previously described.[^32^](#_ENREF_32)^,^[^33^](#_ENREF_33)

**Statistical Analysis**

R 3.0 (R-project.org), Graphpad Prism 5.0 (GraphPad Software Inc; La Jolla, CA, USA), and Excel 2010 (Microsoft; Richmond, WA, USA) software packages were used to calculate mean values, standard deviations, and p-values using the t-test model, Mann-Whitney U model, chi-square test or fitting linear model as stated. Differences in protein expression among subgroups were analyzed with Image J (National Institutes of Health, USA). For all tests, a significant p-value < 0.05 is represented with *, a p-value of < 0.01 is represented with **, and p < 0.001 is represented with ***. Error bars represent the standard deviation.
